# Humanization of high-affinity antibodies targeting glypican-3 in hepatocellular carcinoma

**DOI:** 10.1038/srep33878

**Published:** 2016-09-26

**Authors:** Yi-Fan Zhang, Mitchell Ho

**Affiliations:** 1 Antibody Therapy Section, Laboratory of Molecular Biology, Center for Cancer Research, National Cancer Institute, National Institutes of Health, Bethesda, MD 20892, United States

## Abstract

Glypican-3 (GPC3) is a cell-surface heparan sulfate proteoglycan highly expressed in hepatocellular carcinoma (HCC). We have generated a group of high-affinity mouse monoclonal antibodies targeting GPC3. Here, we report the humanization and testing of these antibodies for clinical development. We compared the affinity and cytotoxicity of recombinant immunotoxins containing mouse single-chain variable regions fused with a *Pseudomonas* toxin. To humanize the mouse Fvs, we grafted the combined KABAT/IMGT complementarity determining regions (CDR) into a human IgG germline framework. Interestingly, we found that the proline at position 41, a non-CDR residue in heavy chain variable regions (VH), is important for humanization of mouse antibodies. We also showed that two humanized anti-GPC3 antibodies (hYP7 and hYP9.1b) in the IgG format induced antibody-dependent cell-mediated cytotoxicity and complement-dependent-cytotoxicity in GPC3-positive cancer cells. The hYP7 antibody was tested and showed inhibition of HCC xenograft tumor growth in nude mice. This study successfully humanizes and validates high affinity anti-GPC3 antibodies and sets a foundation for future development of these antibodies in various clinical formats in the treatment of liver cancer.

Glypican-3 (GPC3) is a glycophosphatidylinositol (GPI)-anchored cell surface heparan sulfate proteoglycan that is expressed during early development, and expression can be detected in human embryo, fetus and placental tissues^1^, but not in normal adult tissue^2^. GPC3 overexpression is associated with liver cancers, including hepatocellular carcinoma (HCC)^3^ and hepatoblastoma^4^. GPC3 is involved in HCC tumorigenesis through Wnt^5^,^6^,^7^, Yap^8^, TGF-β2^9^ and HGF^10^ signaling. Its oncofetal expression and role as an important signaling modulator suggest that GPC3 could be a potential therapeutic target in cancer^11^.

We generated a series of high affinity anti-GPC3 mouse monoclonal antibodies (YP7, YP8, YP9, YP9.1) by immunizing mice with a C-terminal peptide derived from human GPC3 isoform 2 (Residues: 511–560)^12^. We initially tested one of these antibodies (YP7) and found that it had very specific binding towards HCC tumor cells in patient tissues and inhibited the growth of a hepatoblastoma xenograft tumor in nude mice^12^. Here we report the sequences of these mouse variable regions (Fvs). We compared the affinity and cytotoxicity of these mouse single-chain Fvs fused to a bacterial toxin in order to select the candidates with which to move forward to humanization.

One issue facing all antibody-based therapeutics is the activation of secondary immune responses to foreign proteins. One proven method for reducing the immunogenicity is to humanize the antibody. Grafting the complementarity determining region (CDR) is a widely-used method to humanize the antibodies^13^,^14^. The CDR identified by Kabat *et al*. is based on the sequence variability of human, mouse and rabbit antibodies^15^,^16^, whereas the IMGT CDR takes into account both the sequence variability calculated by Kabat and the antibody structure or antibody-antigen structure complex^17^. We recently humanized a rabbit monoclonal antibody (YP218) by grafting dual CDRs (KABAT and IMGT) to the most similar human germline sequence without the need for back-mutation^18^. Here, we tested whether the same dual CDR grafting method could be used to humanize the mouse anti-GPC3 antibodies. Interestingly, we found a non-CDR residue, the proline at position 41 in VH, is important in humanization of mouse antibodies and should be retained during humanization for the best activity and antigen binding affinity. Our humanized antibodies (hYP7 and hYP9.1b) retained high functional binding affinity and induced antibody-dependent cell-mediated cytotoxicity (ADCC) and complement-dependent cytotoxicity (CDC). Furthermore, we found that the hYP7 antibody inhibited Hep3B (an HCC cell line) xenograft tumor growth in nude mice.

## Results

### Cloning and sequence analysis of mouse Fvs

**Figure 1:**
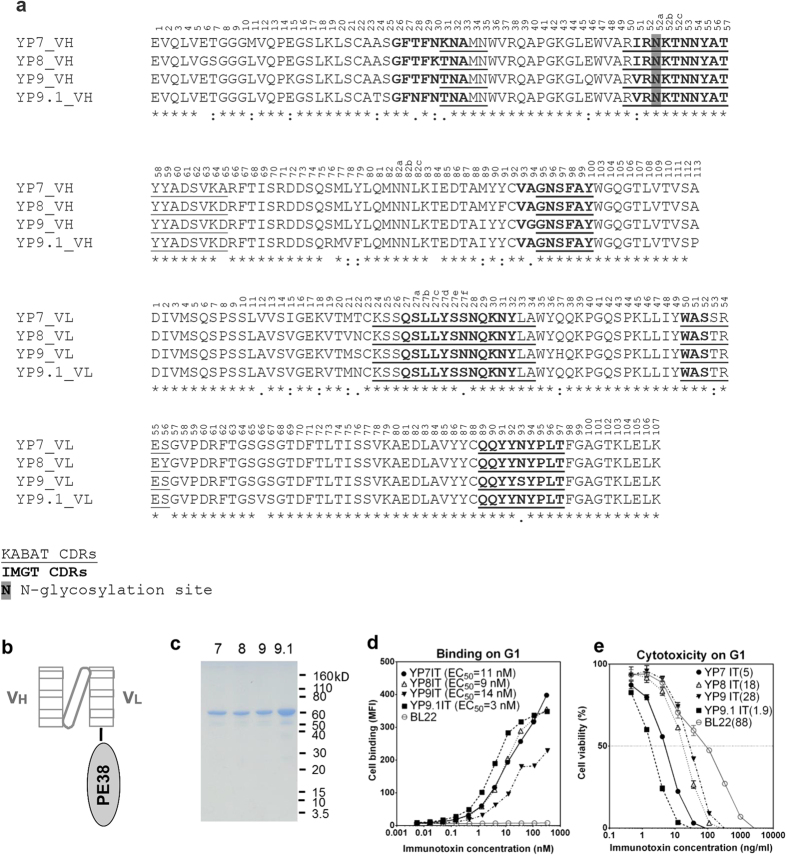
Sequence comparison, binding affinity and cytotoxicity of mouse anti-human GPC3 antibody/immunotoxins. (**a**) Alignment of antibody Fv amino acid sequence of YP7, YP8, YP9 and YP9.1. The numbers reflect the KABAT system. (**b**) The immunotoxin contains the scFv fused to the N-terminus of a truncated *Pseudomonas* exotoxin (PE38). (**c**) SDS-PAGE of purified immunotoxins (IT) of YP7, YP8, YP9 and YP9.1. (**d**) Binding of immunotoxins on G1 cells. (**e**) Cytotoxicity of immunotoxins on G1 cells. IT, immunotoxin. The numbers in parentheses are EC_50_ values of immunotoxins in ng/ml.

To analyze the antigen-binding sequences of our anti-GPC3 antibodies, we cloned the antibody Fv sequences using 5′RACE-PCR from hybridoma cells. Sequencing of the mouse anti-GPC3 monoclonal antibodies revealed that the Fv regions of YP7, YP8, YP9 and YP9.1 were homologous to each other (Fig. 1a). The single-chain Fv (scFv) fragment of each mouse antibody was fused to a truncated version of *Pseudomonas* exotoxin A (PE38) to generate a recombinant immunotoxin (Fig. 1b). These proteins were produced in *E*. *coli* and their purities were checked by SDS-PAGE (Fig. 1c). To determine the binding efficiency and cytotoxicity of the newly constructed immunotoxins (IT), we used a G1 cell line, an A431 line that overexpresses the GPC3 protein^12^, to determine binding efficiency and cytotoxicity (Fig. 1c,d). YP9.1 immunotoxin (YP9.1IT) was found to have the highest affinity (EC_50_ = 3 nM) and cytotoxicity (EC_50_ = 1.9 ng/ml). YP7IT and YP8IT had similar avidities (EC_50_ at around 10 nM), but YP7IT had much stronger cytotoxicity (EC_50_ = 5 ng/ml) than YP8IT (EC_50_ = 18 ng/ml). YP9IT had the lowest affinity and cytotoxicity. It is noted that the only N-glycosylation motif is within the VH CDR2 (residue 52a) of these antibodies (Fig. 1a), but it does not seem to affect the activity as their binding was confirmed in the format of the bacteria-expressed immunotoxins which do not have N-glycosylation.

### Humanization of Fvs via dual CDR grafting

**Figure 2:**
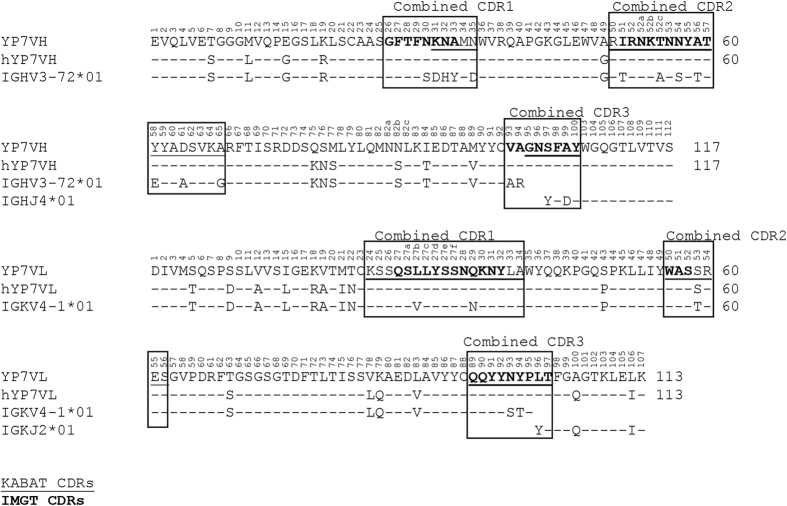
Alignment of YP7 and humanized YP7 (hYP7) Fv sequence with its grafting template. The numbers reflect the KABAT system.

**Figure 3:**
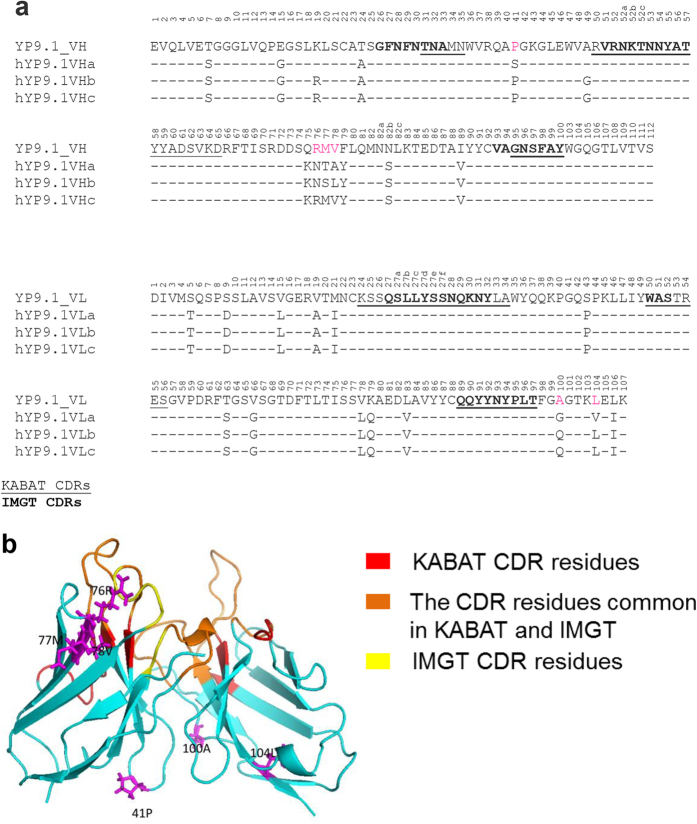
Humanization of the YP9.1 antibody. (**a**) Alignment of Fv sequence of YP9.1 and the three versions of humanized YP9.1. The numbers reflect the KABAT system. (**b**) The structure model of YP9.1 generated by Rosetta (provided by ROSIE Server). The KABAT CDR residues are shown in red; the IMGT residues are shown in yellow. The CDR residues common in KABAT and IMGT are shown in orange. The 41P and 76RMV in VH, 100A and 104L in VL are shown in magenta.

**Figure 4:**
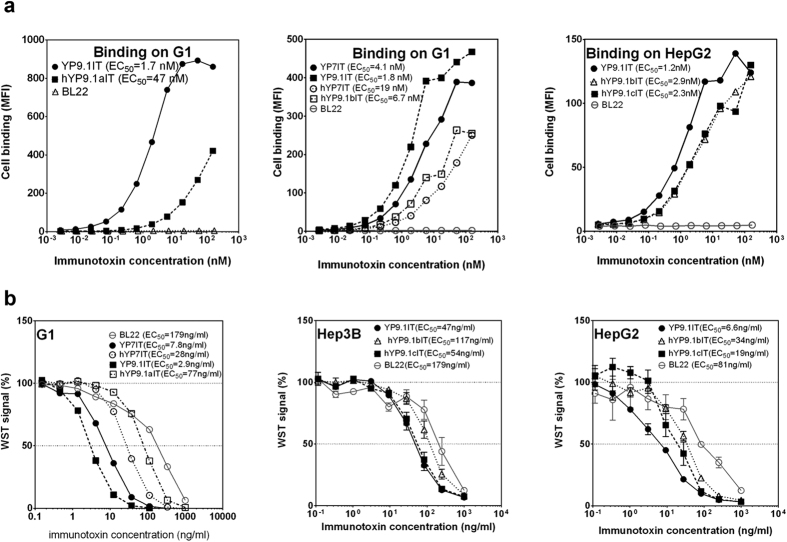
The affinity and cytotoxicity measurement of anti-GPC3 immunotoxins. (**a**) The binding of immunotoxin on GPC3+ cells. (**b**) The cytotoxicity of immunotoxins on cells. IT, immunotoxin.

To humanize anti-GPC3 antibodies for potential clinical development, the YP7 and YP9.1 murine Fvs were selected because of their high affinity and cytotoxicity (Fig. 1d,e). We constructed humanized YP7 (hYP7) by grafting dual CDRs (KABAT/IMGT) onto its most similar human germline sequences (Fig. 2). The first version of YP9.1 (hYP9.1a) was similarly humanized, but the dual CDRs were grafted to different germline sequences due to higher level of sequence homology (Fig. 3a). To compare with the original Fvs, we constructed the immunotoxins based on humanized Fvs and evaluated their binding avidities (Fig. 4a) and cytotoxicity (Fig. 4b). The EC_50_ of hYP7 immunotoxin (hYP7IT) was 19 nM in G1 cells, which was only 4.6-fold higher than the original EC_50_ of YP7IT (Fig. 4a), whereas the EC_50_ of the resulting hYP9.1 immunotoxin (hYP9.1aIT) was 28-fold higher than the original EC_50_ of YP9.1IT, indicating that the hYP7IT framework retains the binding affinity better than the framework of hYP9.1aIT.

We also humanized YP9.1 using the framework of hYP7. The EC_50_ of the resulting “b” version (hYP9.1bIT, EC_50_ = 6.7 nM)) was only 3.7-fold higher than the original EC_50_ of YP9.1IT (EC_50_ = 1.8 nM). As shown in Fig. 3, hYP9.1bIT kept the following mouse sequences that hYP9.1aIT changed: VH residue 41 (hYP9.1aIT changed from P to S), VL residue 104 (hYP9.1aIT changed from L to V); Fig. 3 also shows that the hYP9.1bIT and hYP9.1aIT have different mutations for the following amino acids: VH residue 77 and 78 (original is MV, hYP9.1aIT is TA, hYP9.1bIT is SL), and VL residue 100 (original is A, hYP9.1aIT is G, hYP9.1bIT is Q). Among these sites, only VH residues 77 and 78 are close to the antigen binding site (Fig. 3b). To test whether 76RMV78 in VH contributes to antigen binding and the much higher affinity of YP9.1, and to generate hYP9.1 with higher affinity, we generated another version of hYP9.1 by combining two human VH sequences as the framework to retain only 76RMV78. The resulting “c” version (hYP9.1cIT) had almost the same affinity as hYP9.1bIT in HepG2 cells (Fig. 4a), indicating that this change was not important. Therefore, we decided to use YP9.1bIT in the rest of our study. Using IMGT/domainGapAlign, the percentages of identical residues to the most similar human germline variable sequence were 87.8% and 95% for hYP7 VH and VL, respectively, and 86.7% and 96% for hYP9.1b VH and VL, respectively.

The change in cytotoxicity of these immunotoxins correlated well with the change in their binding affinity after humanization (Fig. 4b). The hYP7IT exhibited a 3.6-fold reduction in cytotoxicity against G1, hYP9.1aIT lost 26-fold of cytotoxicity against G1, hYP9.1bIT lost 2.5-fold cytotoxicity against Hep3B and 5.2-fold cytotoxicity against HepG2, similar to the observed affinity loss. Although remaining the VH 76RMV78 in hYP9.1cIT did not improve the cell binding, it slightly improved the cytotoxicity of the immunotoxin: the hYP9.1cIT had similar cytotoxicity as the original YP9.1bIT against Hep3B, and lost only 2.9-fold of cytotoxicity against HepG2.

### Evaluation of humanized antibodies to GPC3 in IgG format *in vitro*

**Figure 5:**
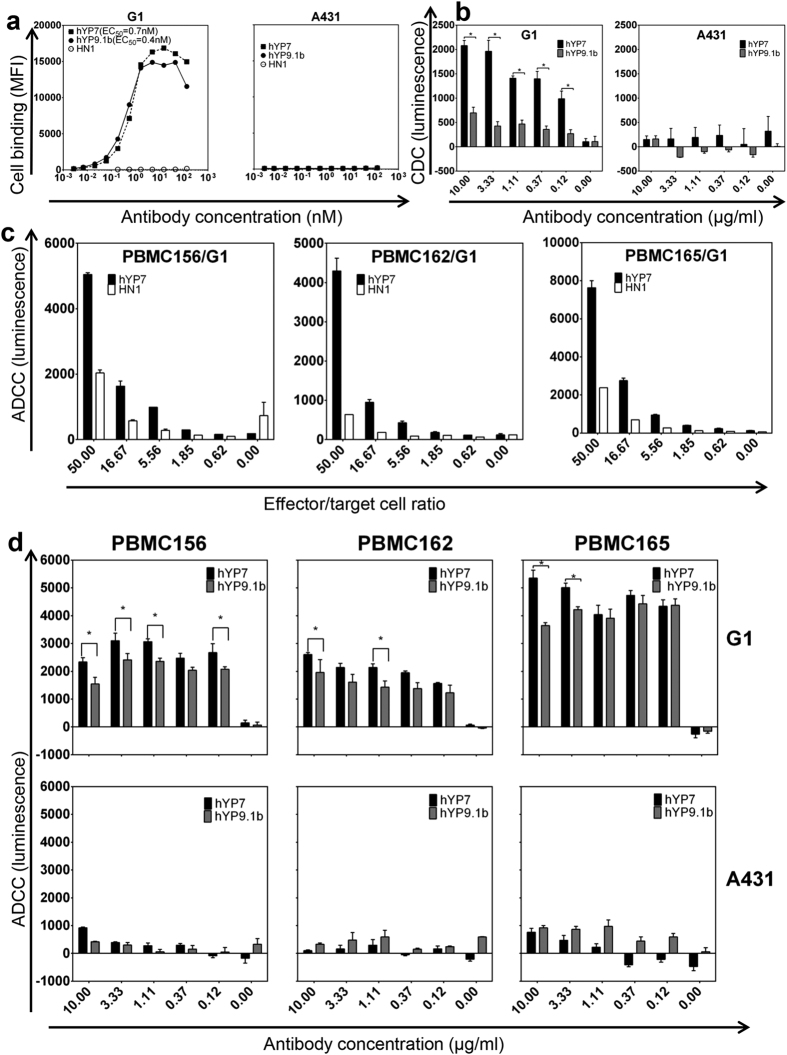
The affinity, antibody-dependent cell-mediated cytotoxicity (ADCC) and complement-dependent cytotoxicity (CDC) measurement of anti-GPC3 antibodies (IgG1). (**a**) Binding of hYP7 and hYP9.1b (all in IgG format) on cells. A431 cells are GPC3 negative. (**b**) CDC assays of anti-GPC3 antibodies. (**c**) ADCC assays with various effector:target cell ratios. The target cells have been preincubated on ice with 1 μg/ml antibody. HN1 is the negative control. PBMCs 156, 162 and 165 are PBMCs isolated from three healthy donors. (**d**) ADCC assays with various antibody concentrations and at an effector:target cell ratio of 25:1. The luminescence from the wells without antibodies was treated as background and subtracted in panels (b,d). The luminescence from the wells with control antibodies at indicated concentrations was treated as background and subtracted. A431 cell is GPC3-, and G1 cell is A431 cell overexpressing GPC3. Asterisk (*) indicate statistical significance (p < 0.05, tested by t test).

To evaluate humanized antibodies in the IgG format, we fused antibody Fv sequences of hYP7 and hYP9.1b to human immunoglobulin γ1 and κ constant regions and expressed them in HEK 293T cells. We compared their EC_50_ values in GPC3+ cells (G1) and GPC3- (A431) cells. The hYP7 and hYP9.1b antibodies had similar binding EC_50_ on G1, with EC_50_ at 0.7 nM and 0.4 nM, respectively. All of them did not stain GPC3 negative A431 cells even at high concentrations (Fig. 5a), indicating their binding was highly specific for cell surface GPC3.

We then examined effect upon ADCC and CDC as a result of antibody binding in G1 cells (GPC3+) and A431 cells (GPC3-) stably expressing luciferase. Both hYP7 and hYP9.1b induced CDC in G1, but not in A431 (Fig. 5b). The hYP7 antibody had better CDC activity than hYP9.1b. For the ADCC assay, we used human peripheral blood mononuclear cells (PBMC) isolated from three different healthy donors, and found all of them killed the G1 cells. Increasing the effector/target cell ratios caused an increase in the cytotoxicity (Fig. 5c). Both hYP7 and hYP9.1b antibodies induced specific ADCC in G1 at a concentration as low as 0.12 μg/ml, but not in the GPC3- cell line A431 (Fig. 5d). Taken together, the hYP7 antibody is more potent than hYP9.1b in both ADCC and CDC assays. Therefore, the hYP7 antibody was selected for mouse testing.

### Evaluation of hYP7 in mice

**Figure 6:**
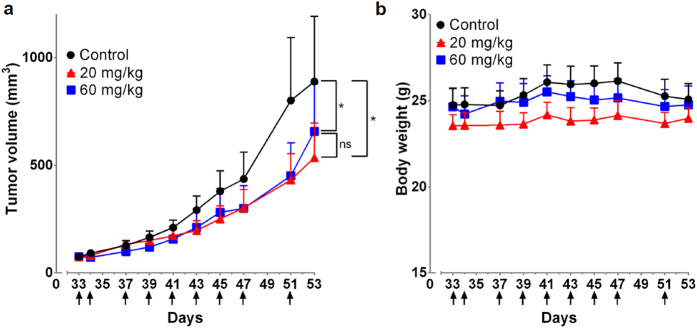
*In vivo* antitumor activities of hYP7. (**a**) The *in vivo* inhibition of hepatocellular carcinoma xenograft tumor growth by hYP7 in nude mice. The 20 mg/kg group and the 60 mg/kg group showed significantly slower tumor growth than the control (p < 0.05, tested by paired t test). There were no statistically significant differences (ns) in tumor growth between 20 mg/kg group and 60 mg/kg group when tested with the same method. (**b**) Body weight measurements. Arrows indicated individual injections; n = 6/group. Values represent mean ± s.e.m.

To determine the effect of hYP7 on *in vivo* cell killing, we used a Hep3B HCC xenograft tumor model in nude mice. The hYP7 IgG was administered over nine doses at 20 mg/kg and 60 mg/kg. Treatment began on day 33 when the average tumor volume reached 75 mm^3^. By day 53 the tumor size for the 20 mg/kg and 60 mg/kg were 60% and 74% of the control tumor, respectively, and the growth differences between the control and each of the two treated groups were statistically significant (Fig. 6a). Tumor growth rate between the two treatment groups were not statistically significant (Fig. 6a), and we did not observe a significant loss in body weight during or after treatment (Fig. 6b).

## Discussion

In the present study, we have successfully humanized and validated high affinity anti-GPC3 monoclonal antibodies. The mouse anti-human GPC3 C-peptide antibodies YP7, YP8, YP9 and YP9.1 have very similar VH and VL sequences, suggesting that they target a similar epitope. However, the minor difference in sequence greatly affected their affinity.

We recently humanized a rabbit monoclonal antibody by grafting the dual KABAT/IMGT CDR to a human germline framework without back mutation to original sequence^18^. Here, we explored whether a similar strategy could be applied to the humanization of mouse antibodies for clinical development. Surprisingly, we found a significant difference in binding avidities between two versions of humanized YP9.1: hYP9.1aIT and hYP9.1bIT. We found four sites of hYP9.1bIT that could potentially account for its higher affinity than hYP9.1aIT: VH residue 41, VL residue 104, VH residue 77 and 78, and VL residue 100. The site 76RMV78 is located close to the heavy chain FR-3 loop, and often directly contacts the antigen^19^ or important residues (such as residue 71 in VH and VL^20^,^21^, and residues H72 and H75 in the FR-3 loop^21^). However, experiments comparing hYP9.1bIT with hYP9.1cIT, which retains 76RMV78 in the mouse sequence, revealed no significant difference in binding affinities suggesting that this region is likely not essential for binding in this case. Among other differences, the change at VH position 41 from P to S in hYP9.1aIT appears significant. Proline and serine are structurally different, and the 41P positioned in the FR-2 loop, which is involved in heavy chain-light chain interaction^22^,^23^ and undergoes more conformation change upon antibody binding than other residues in the FRs^24^. Between 2009 and November 2015, we found 25 mouse antibody humanization studies in PubMed (Table 1) with eighteen of these mouse antibodies containing 41P and their humanized versions do retain 41P. For the 7 mouse antibodies that do not have 41P, their humanized versions all changed into 41P. Compared to VH 41P, we suspect the other two changes are less likely to be important for YP9.1 binding. The L to V change in YP9.1aIT adds only a single CH2 group to the side chain, and is located (position 104 in VL) far from the antigen binding site. The A to G changes in hYP9.1aIT at VL position 100 is smaller than the A to Q change in hYP9.1bIT at the same position and is less likely to be the reason for the improved binding in hYP9.1bIT. Based on our data and other studies, we speculate that the 41P non-CDR residue in VH is important and should be retained during the humanization of mouse antibodies for the best activity.

The therapeutic potential of hYP7 and hYP9.1b (IgG1κ) was evaluated in ADCC and CDC assays *in vitro*. In both assays, we measured the luciferase activity in culture supernatant as an indication of cell death. Our results demonstrated that hYP7 is more effective in inducing ADCC and CDC in G1 cells when compared with hYP9.1b. The luciferase assay has low background because only dead cells release luciferase. However, unlike the ^51^Cr-release assay^25^, the luciferase assay has its limitations because luciferase activity decays during prolonged incubation in culture supernatant. Therefore, this assay is more suitable to compare different antibodies than to determine the % cytotoxicity induced by ADCC or CDC.

The therapeutic potential for naked hYP7 was also tested in an HCC xenograft model in nude mice. However, we only saw a moderate therapeutic effect. Previously we showed that the murine version of YP7 had more potent anti-tumor activity in mice^12^, a finding which we believe may be due to the humanized version triggering the formation of anti-human antibodies in nude mice^26^. The only anti-GPC3 antibody in clinical trial, GC33 did not show a clinical benefit in randomized phase 2 trials^27^. The clinical data indicates that the naked antibody format is not a promising therapeutic approach for liver cancer. In our mouse studies with hYP7, we did not observe a statistical difference between the 20 mg/kg and 60 mg/kg group, suggesting that 20 mg/kg dose fully covered the exposed GPC3 on the tumor cell surface. Clearly, the limiting factor for therapeutic effect was not the dose, but rather, more specifically due to the intrinsic character of the naked antibody format. On the contrary, our previous study showed that the immunotoxin format worked much better than the naked antibody^7^. Our anti-GPC3 heavy-chain antibody, HN3, showed much better therapeutic effect in its immunotoxin format than in its naked antibody format, and the HN3 immunotoxin further regressed the tumor^7^,^8^.

In conclusion, we have humanized YP7 and YP9.1 anti-GPC3 Fvs with high affinity by retaining dual CDRs (KABAT and IMGT) and key non-CDR residues, in particular 41P. It would be interesting to explore whether our strategy could be applicable to humanization of other mouse monoclonal antibodies. Given the strength and specificity of their binding, hYP7 and hYP9.1b can potentially be developed for clinical applications. Based on our data and others^28^, naked anti-GPC3 antibodies do not have curative treatment of liver cancer in mice and humans although the antibodies have excellent binding affinity and specificity for GPC3-positive liver cancer cells. Therefore, it would be interesting to construct and evaluate chimeric antigen receptors (CARs)^29^, antibody-drug conjugates (ADCs)^30^ and bispecific antibodies^31^ using anti-GPC3 antibodies for future clinical development. These antibodies can potentially be made into *in vivo* diagnostic tools, too, such as *in vivo* tumor imaging and fluorescence-guided surgery^32^.

## Materials and Methods

### Cell cultures

Mouse hybridomas were cultured in a medium containing 80% DMEM, supplemented with 20% fetal bovine serum (FBS), 2% L-glutamine, 1% non-essential amino acid (NEAA), 1% HEPES and 1X hypoxanthine-aminopterin-thymidine (HAT) selection media (Cellgro, VA). Hep3B and HepG2 cells (purchased from ATCC, Manassas, VA) were cultured in 90% DMEM, supplemented with 10% FBS, 1X GlutaMAX and 1X Penicillin/Streptomycin. Cells were grown at 37 °C and 5% CO_2_. G1 is a transfected A431 human epithelial carcinoma cell line produced in our lab that stably expresses human GPC3^12^. G1 or A431 cells were stably transfected with a luciferase gene (kindly provided by Lentigen Technology Inc, Gaithersburg, MD) and were cultured in the same medium as described^12^.

### Cloning of antibody Fv sequences

The mouse antibody Fv sequences were cloned using 5′RACE with modified primers and conducted as described previously^33^,^34^. The primers are listed in Table 2. To prepare cDNA templates, mRNA was extracted from hybridoma cells with Illustra QuickPrep Micro mRNA Purification Kit (GE Healthcare, Buckinghamshire, UK). Five hundred nanograms of mRNA were reverse-transcribed into first strand cDNA with SuperScript III First-Strand Synthesis Supermix (Life Technologies, Grand Island, NY). The reaction mix was then treated with RNase H (NEB, Ipswich, MA) and processed for PCR purification (QIAquick PCR purification kit, Qiagen). Poly-dC was added by terminal transferase (NEB) to the 3′ end of the first strand cDNA (corresponding to the 5′ end of the original mRNA). The products were purified with a QIAquick PCR purification kit again before PCR reactions. The 5′RACE was performed using Phusion Hot Start II High-Fidelity DNA Polymerase (Thermo Scientific, Cincinnati, OH). The Fv fragments were cloned into the pCR4-TOPO vector with a TOPO TA Cloning Kit (ThermoFisher Scientific) for sequencing according to manufacturer’s instructions. Positive clones were screened with Taq DNA polymerase. BigDye (Invitrogen) was used for sequencing according to manufacturer’s instructions.

### Humanization of anti-GPC3 antibodies

Mouse YP7 and YP9.1 antibodies were humanized using a dual CDR-grafting method, as described in our previous report about humanization of a rabbit monoclonal antibody^14^. The VH and VL sequences were searched against the human germline sequence databases with IgBLAST (http://www.ncbi.nlm.nih.gov/igblast/) and IMGT/V-QUEST (http://www.imgt.org/IMGT_vquest/share/textes/), and the most similar human germ line Fv sequence and J region were identified. The residues within either KABAT or IMGT CDR regions were grafted onto the framework regions of templates. The antibody structure model was built with either WAM (http://antibody.bath.ac.uk/) or ROSIE server^35^,^36^.

### Immunotoxin production and testing

The immunotoxins were constructed, produced and tested for cell binding and cytotoxicity as described previously^18^,^37^. The scFv fragments were cloned into pRB98 vector to make pMH151 (YP7), pMH153 (YP9), pMH154 (YP9.1), pMH170 (hYP7), pMH171 (hYP9.1a) and pMH172 (hYP9.1b) plasmids. The cell proliferation was measured as WST signals; higher signals correspond to more viable cells.

### Production of humanized IgG antibodies

The humanized VH and VL sequences were fused to human Fc fragments in the pDR12 backbone vector^38^ to make pMH178 (hYP7) and pMH180 (hYP9.1) plasmids. For *in vitro* testing, the antibodies were produced in HEK 293T cells by transient transfection with polyethylenimine (PEI 25 Kd linear, Polysciences, Warrington, PA). The secreted humanized IgG in the culture supernatant was purified by affinity chromatography with a protein A column (GE). For animal testing, the hYP7 IgG was produced by Novoprotein (Summit, NJ).

### Flow cytometry

Various concentrations of antibodies were incubated with suspended cells in a 96-well plate at 4 °C for 1 hour. Bound antibodies were labeled with either R-phycoerythrin conjugated goat anti-mouse IgG (Southern Biotech) or R-Phycoerythrin-conjugated F(ab’)2 goat anti-human IgG Fc (Jackson ImmunoResearch). Cells were analyzed with FACSCalibur (BD Biosciences, San Jose, CA) and the data were analyzed with FlowJo (Tree Star, Inc., Ashland, OR) and GraphPad Prism (GraphPad Software, Inc., La Jolla, CA).

### Antibody-dependent cell-mediated cytotoxicity (ADCC) and complement dependent cytotoxicity (CDC) assay

The ADCC and CDC were measured using luciferase release assays using A431 cells (GPC3-) and G1 cells (GPC3+) stably transfected with a luciferase gene. The lysed cells release luciferase into the culture supernatant, which was quantified by luciferase activity. For ADCC assays, the human peripheral blood mononuclear cells (PBMC) were purified from buffy coat obtained from three healthy donors (Oklahoma Blood Institute, Oklahoma City, OK). All the experiments involving human subjects were carried out in accordance with the approved guidelines. Informed consent was obtained from all subjects. Briefly, the buffy coat were diluted with equal volumes of PBS, and then layered over 15 ml Ficoll-Paque plus (GE Healthcare, Pittsburgh, PA) in a 50 ml conical tube. The tubes were then spun at 140 g for 10 min at room temperature in a swing-bucket centrifuge, then at 670 g for 15 min. PBMC at the interface between aqueous layer and the Ficoll were removed and washed with PBS four times in a 50 ml tube by centrifuging at 250 g for 5 min each. The PBMC, target cells and antibodies (hYP7, hYP9.1b and an irrelevant negative control antibody HN1^39^) were mixed at indicated ratios or concentrations (Fig. 5) when seeded into the 96 well plates. They were incubated in a 37 °C incubator with 5% CO_2_ overnight before measurements. For CDC assays, we pre-incubated target cells with 1.7x indicated concentrations (Fig. 5b) of antibodies (hYP7, hYP9.1b and an irrelevant control negative antibody HN1^39^) in 96 plates at 4 °C, and then add pooled complement human serum (Innovative Research, Novi, MI) into each well to make the final concentration of human serum at 20% and the antibody concentrations 1x. They were incubated in a 37 °C incubator with 5% CO_2_ for 2 hours before measurements. In both assays, we seeded 10,000 target cells per well. To measure the luciferase activity as a quantification of cell death, 10 μl of supernatant from each well were mixed with 50 μl of luciferase assay reagent prepared according to the manufacturer’s instruction (Promega), and the luminescence was measured with a Victor^3^ 1420 multilabel counter (PerkinElmer, Waltham, MA). The ADCC and CDC were measured as the luminescence subtracted with luminescence from control antibody treated wells.

### Animal and tumor studies

All mice were housed and treated under the protocol (LMB-059) approved by the Institutional Animal Care and Use Committee at the National Institutes of Health (NIH). The animal experiments were performed in accordance with the approved guidelines. To make the xenograft tumor model, 5 × 10^6^ Hep3B cells were suspended in 200 μl of PBS and inoculated subcutaneously (s.c.) into 5-week-old female BALB/c nu/nu nude mice (NCI- Frederick Animal Production Area, Frederick, MD). Tumor dimensions were determined using calipers and tumor volume (mm^3^) was calculated by the formula V = ab^2^/2, where a and b represent tumor length and width, respectively. When the average tumor size reached ~75 mm^3^, the mice were intravenously injected with indicated dose of hYP7 on the indicated dates.

## Additional Information

**How to cite this article**: Zhang, Y.-F. and Ho, M. Humanization of high-affinity antibodies targeting glypican-3 in hepatocellular carcinoma. *Sci. Rep.* **6**, 33878; doi: 10.1038/srep33878 (2016).

## Figures and Tables

**Table 1:** A summary of 41P in mouse antibody humanization literature between 2009 and November 2015.

Mouse Ab	Target	Closest mouse VH germline	Original 41P	Retained 41P	Convert to 41P	References
8H9	B7-H3	IGHV1-85*01	1	1		40
5F9	CD47	IGHV1-12*01	1	1		41
KR127	preS1 (HBV)	IGHV1-82*01	1	1		42
Rituximab	CD20	IGHV1-12*01	1	1		43
MR1	EGFRvIII	IGHV5-6*03	0		1	44
30D8	CXCL12	IGHV2-6-4*01	1	1		45
AD11	hβNGF	IGHV2-6-7*02	0		1	46
D9	Ricin	IGHV1-26*01	0		1	47
ICR62	EGFR	IGHV1-22*01	0		1	48
1567	CCR4	IGHV1S56*01	1	1		49
3F8	GD2	IGHV2-9*02	1	1		50
αD11	hβNGF	IGHV2-6-7*02	0		1	a different humanization version of AD11 51
14F7	ganglioside	IGHV1-7*01	1	1		52
3G8	CD16	IGHV8-8*01	0		1	31
m357	TNF-α	IGHV6-3*01	1	1		53
LD1	FGF receptor 4	IGHV1-61*01	1	1		54
muAb2/3H6	human monoclonal antibody 2F5 antigen binding site	IGKV17-121*01	1	1		55
m836	IL-13	IGHV8-8*01	0		1	56
tumex	Membrane-Associated Heat Shock Protein 70	IGHV2-6-4*01	1	1		57
AY4	death receptor 4	IGHV5-6-5*01	1	1		58
m9O12	platelet glycoprotein VI	IGHV1-12*01	1	1		59
5S	Hepatitis B surface antigen	IGHV1-12*01	1	1		60
4G7	CD19	IGHV1-14*01	1	1		61
3D8	DNA	IGHV1-14*01	1	1		62
muA9	CD16	IGHV1-63*02	1	1		63

**Table 2:** Primers used in 5′RACE.

**Primer’s name**	**Sequence**	**Usage**
MG1-Hinge_708-683R	ACCACAATCCCTGGGCACAATTTTCT	RT for IgG1 heavy chain
MG1-PCR_438-409R	AGGGGCCAGTGGATAGACAGATGGGGGTGT	1st PCR for IgG1 heavy chain
MVH2R	ATAGACAGATGGGGGTGTCGTTTTGGC	2nd PCR for IgG1 heavy chain
MK-Edge_711-685R	CTCATTCTTGTTGAAGCTCTTGACAAT	RT for Kappa chain
MK-PCR_435-403R	GGATGGTGGGAAGATGGATACAGTTGGTGCAGC	1st PCR for Kappa chain
Adaptor + G	GGCCACGCGTCGACTAGTACGGGGGGGGGG	1st PCR
Adaptor	GGCCACGCGTCGACTAGTAC	2nd PCR

RT: reverse transcript.
